# Changing relative risk of clinical factors for hospital-acquired acute kidney injury across age groups: a retrospective cohort study

**DOI:** 10.1186/s12882-020-01980-w

**Published:** 2020-08-02

**Authors:** Lijuan Wu, Yong Hu, Xiangzhou Zhang, Weiqi Chen, Alan S. L. Yu, John A. Kellum, Lemuel R. Waitman, Mei Liu

**Affiliations:** 1 Big Data Decision Institute (BDDI), Jinan University, Guangzhou, 510632 China; 2 Guangdong Engineering Technology Research Center for Big Data Precision Healthcare, Guangzhou, 510632 China; 3 Division of Nephrology and Hypertension and the Kidney Institute, University of Kansas Medical Center, Kansas City, 66160 USA; 4 Center for Critical Care Nephrology, Department of Critical Care Medicine, University of Pittsburgh School of Medicine, Pittsburgh, 15260 USA; 5 Department of Internal Medicine, Division of Medical Informatics, University of Kansas Medical Center, Kansas City, 66160 USA

**Keywords:** Acute kidney injury (AKI), Risk factor, Risk prediction, Electronic medical record, Machine learning

## Abstract

**Background:**

Likelihood of developing acute kidney injury (AKI) increases with age. We aimed to explore whether the predictability of AKI varies between age groups and assess the volatility of risk factors using electronic medical records (EMR).

**Methods:**

We constructed a retrospective cohort of adult patients from all inpatient units of a tertiary care academic hospital and stratified it into four age groups: 18–35, 36–55, 56–65, and > 65. Potential risk factors collected from EMR for the study cohort included demographics, vital signs, medications, laboratory values, past medical diagnoses, and admission diagnoses. AKI was defined based on the Kidney Disease Improving Global Outcomes (KDIGO) serum creatinine criteria. We analyzed relative importance of the risk factors in predicting AKI using Gradient Boosting Machine algorithm and explored the predictability of AKI across age groups using multiple machine learning models.

**Results:**

In our cohort, older patients showed a significantly higher incidence of AKI than younger adults: 18–35 (7.29%), 36–55 (8.82%), 56–65 (10.53%), and > 65 (10.55%) (*p* < 0.001). However, the predictability of AKI decreased with age, where the best cross-validated area under the receiver operating characteristic curve (AUROC) achieved for age groups 18–35, 36–55, 56–65, and > 65 were 0.784 (95% CI, 0.769–0.800), 0.766 (95% CI, 0.754–0.777), 0.754 (95% CI, 0.741–0.768), and 0.725 (95% CI, 0.709–0.737), respectively. We also observed that the relative risk of AKI predictors fluctuated between age groups.

**Conclusions:**

As complexity of the cases increases with age, it is more difficult to quantify AKI risk for older adults in inpatient population.

## Background

Acute kidney injury (AKI) is a common and highly lethal clinical problem, affecting 11–12% of all hospitalized patients worldwide with a mortality rate of ~ 10% [1]. AKI is associated with significant short- and long-term morbidity and mortality [2], and prevention is the best means for dealing with AKI. Delays in identification and intervention for AKI may lead to rapid progression of the kidney injury, likelihood of developing chronic kidney disease (CKD), need for renal replacement therapy, and risk of death [3]. Hence, AKI risk assessment and management based on susceptibilities and exposures are recommended by the Kidney Disease Improving Global Outcomes (KDIGO) guidelines as it may trigger early effective interventions such as drug dose adjustment, avoidance of nephrotoxins, and intravenous fluids management [4]. Early subspecialist (nephrologist, intensivist) or pharmacist involvement in the care of AKI patients can reduce the risk of further kidney function decline [5].

AKI is associated with various risk factors including inherent factors, exposure to nephrotoxins (e.g. non-steroidal anti-inflammatory drugs [6]), acute illnesses (e.g. sepsis [7]) and major surgeries (e.g. cardiopulmonary bypass or coronary angiography [8–10]). Inherent risk factors include susceptibilities of each individual patient (e.g. age [11]) and those associated with reduced kidney reserve or failure of other organs with known cross-talk with the kidneys (e.g. heart, liver, and respiratory system) [12]. There is strong evidence supporting the role of advanced age in AKI. Elderly patients are at much higher risk for developing AKI due to their decreasing renal reserve and structural changes in the aged kidney that impair its ability to withstand and recover from injury [13].

Primary focus of existing AKI studies has been prediction tools for the early identification of at-risk patients. Studies [11, 14, 15] mainly used a small set of highly correlated risk factors based on existing evidence to build prediction models, which may miss potential unknown risk factors. In addition, there has been significant progress in the applications of machine learning to predict AKI risk using electronic health records (EHR) [16]. Sutherland et al. [17] found that most models had modest predictive success with AUC approximating 0.75. Li et al. applied convolutional neural network to ICU patients achieving an AUC of 0.78. In particular, Tomasev et al. [18] used EHR from the US VA health system to build a deep prediction model achieving an AUC of 0.92 for the 48-h prediction time window.

Existing studies suggest that age can modify the intensity of relationships between other factors and AKI. For instance, Kane-Gill et al. [11] analyzed risk factors of AKI for older patients in intensive care units (ICU), and found that the impact of age was so substantial that other risk factors (e.g. sepsis, hypertension, nephrotoxins) lost their ability to predict AKI risk among patients older than 75 years. However, most previous studies [11, 14, 15] examined age as the main effect and considered its interactions with other risk factors one at a time. Despite higher AKI incidence in older adults, how the predictability of AKI risk changes with age is an unanswered question in the current literature. In this study, we investigated the predictability trend of hospital-acquired AKI across age groups using machine learning algorithms and assessed whether relative importance of risk factors in predicting AKI change across age groups.

## Methods

### Study population

All adult patients (age at visit≥18) admitted to the University of Kansas Health System (a tertiary academic hospital) for 2 days or more from November 2007 to December 2016 were included in this retrospective observational cohort study, which included adult patients from all ICU, surgical, and general wards. From a total of 179,370 encounters, we excluded those samples that lacked necessary data elements required to determine the outcome, that is, less than two serum creatinine measurements; and patients with evidences of moderate or severe kidney dysfunction at admission (estimated Glomerular Filtration Rate (eGFR) less than 60 mL/min/1.73 m^2^ or serum creatinine (SCr) level of > 1.3 mg/dL) were also excluded. eGFR was calculated with the Modification of Diet in Renal Disease (MDRD) equation. The final analysis cohort contained 76,957 encounters.

### AKI definition

We staged AKI for severity according to the SCr-based criteria described in the KDIGO clinical practice guidelines [19] (see Supplementary Table S1). Baseline SCr level was defined as the most recent SCr value within two-day window prior to admission if available; otherwise the first SCr value after admission was used as the baseline. Then all pairs of SCr levels measured between admission and discharge were evaluated on a rolling basis to determine the occurrence of AKI.

### Clinical variables

For each hospital encounter in the final analysis cohort, we extracted time stamped clinical data on demographics, vital signs, medications, laboratory values, past medical diagnoses, and admission diagnoses. This study explored the entirety of the above mentioned EHR data types except for laboratory tests where a selected list of labs that may represent potential presence of a comorbidity correlated with AKI [14] was considered. Details of the 1888 clinical variables considered are available in Table 1. It is important to note that SCr and eGFR were not included as predictive variables because they were used to determine the outcome variable, and we aimed to focus on the contribution of other factors. Laboratory values were categorized as unknown, less than reference normal range, within normal range, or greater than the reference normal range. Patient vital signs were discretized into groups as shown in Supplementary Table S2.

**Table 1:** Clinical variables extracted for the study cohort

Feature Category	# of Variables	Details
**Demographics (Demo)**	3	Age, race, gender;
**Vitals (Vitals)**	5	BMI, diastolic BP, systolic BP, pulse, temperature;
**Lab tests (Lab)**	14	Albumin, ALT, AST, Ammonia, Calcium, BUN, Bilirubin, CK-MB, CK, Glucose, Lipase, Platelets, Troponin, WBC;
**Admission diagnoses (DRG)**	315	University Health System Consortium (UHC) APR-DRG; (e.g. liver transplant, heart &/or lung transplant, etc.)
**Medications (MED)**	1271	All medications are mapped to RxNorm ingredient; (e.g. lithium carbonate, pentostatin, ospemifene, oxybutynin, etc.)
**Medical History (CCS)**	280	ICD9 codes mapped to CCS major diagnoses. (e.g. Nervous system congenital anomalies, other congenital anomalies, etc.)

Drug exposure included inpatient (i.e. dispensed during hospitalization) and outpatient drugs (i.e. medication reconciliation and prior outpatient prescriptions). All medication names were standardized by mapping to RxNorm components. Admission diagnosis, that is, the detailed diagnosis-related group (APR-DRG) of all patients, were collected from the data source of the University Health System Consortium (UHC; http://www.vizientinc.com) in HERON. Patient past medical history was captured as primary diagnoses (ICD-9 codes grouped based on the Clinical Classifications Software (CCS) diagnosis categories by the Agency for Healthcare Research and Quality.

### Data processing and statistical analysis

Only the most recently recorded vitals and lab tests before the AKI prediction point (i.e. 24 h prior to AKI event or last normal SCr for non-AKI cases) were used for each encounter. For vital signs, if no values were available, then the median value across the entire cohort for that variable was imputed [20] (information on missing percentages is available in Supplementary Table S3). Missing values among lab tests were captured as a separate category because information may be contained in the choice to not perform a particular test [14]. Medication exposure was defined as true if it was taken within 7-days before the AKI prediction point. Medical history was defined as true if it occurred before the AKI prediction point. Hence, medical history, medication and admission diagnosis were all binary variables (i.e. presence or absence). Finally, we stratified the cohort into four age groups: 18–35, 36–55, 56–65 and > 65 years.

To analyze the volatility of relative risk and prediction performance associated with AKI across age groups, we implemented the following steps: (a) Feature selection or ranking – applied a multivariate embedded Gradient Boosting Machine (GBM [21]) method to rank individual variables according to its importance in AKI prediction. This step ranked the candidate variables among 1888 features to obtain the top-*k* most important predictors for AKI; (b) Predictive modeling – explored four machine learning methods, i.e. logistic regression, support vector machine (SVM) [2], LogitBoost [22, 23], and random forest [24], to assess the prediction performance across age strata. Area under the receiver operating characteristic curve (AUROC) [25] was calculated as the evaluation metric for prediction performance through a 10-fold cross-validation scheme. To determine stable feature ranking across the 10-folds, we averaged the relative importance weights of variables obtained from each fold. Additionally, to address the imbalanced positive-to-negative class issue (AKI to non-AKI ratio), we implemented an under-sampling strategy that would ensure the same number of samples per class in training the model for each fold but keeping the original class ratio in the test dataset. Under-sampling of training dataset is necessary because skewed samples can mislead machine learning algorithms to favor the majority class, in this case non-AKI samples. For comparison, evaluation strategy without under-sampling was also established for the prediction models. Two-tailed *P* values < 0.05 were used to denote statistical significance for all comparisons. Data extraction and processing were executed using Python 3.7 software with scikit-learn package, and other analysis and graphs were drawn using MATLAB software, version R2017b.

## Results

Of the 76,957 encounters meeting the inclusion and exclusion criteria, any stages of AKI occurred in 7259 (9.43%), and 38,887 (50.53%) were aged 56 years or older. Table 2 illustrates the characteristics of patients by age groups, showing that the incidence of AKI rises with age from 7.29% in the youngest group to 10.55% in the oldest group, and the incidence of AKI in male patients is slightly higher than that of females. Additionally, Table 2 shows that most AKI episodes (namely, AKI onset time in terms of number of days from admission) occurred within a week after admission and there is no significant difference between age groups.

**Table 2:** Demographic characteristics and AKI onset time of patients by age category

Variable	Age 18–35 (***n*** = 12,873)		Age 36–55 (***n*** = 25,197)		Age 56–65 (***n*** = 18,098)		Age > 65 (***n*** = 20,789)	
	AKI	Non-AKI	AKI	Non-AKI	AKI	Non-AKI	AKI	Non-AKI
**Age, years, n (%)**								
n (%)	983 (7.29)	11,890 (92.71)	2222 (8.82)	22,975 (91.18)	1906 (10.53)	16,192 (89.47)	2193 (10.55)	18,596 (98.45)
**Race, n (%)**								
White	660 (67.14)	8038 (67.60)	1537 (69.17)	16,652 (72.48)	1444 (75.76)	13,024 (80.43)	1767 (80.57)	15,463 (83.15)
Black	150 (15.26)	1958 (16.47)	420 (18.90)	3808 (16.57)	264 (13.85)	1926 (11.89)	231 (10.53)	1644 (8.84)
Asian	7 (0.71)	147 (1.24)	12 (0.54)	190 (0.83)	17 (0.89)	112 (0.69)	18 (0.82)	151 (0.81)
Other	121 (12.31)	1792 (15.07)	253 (11.39)	2325 (10.12)	181 (9.50)	1130 (6.98)	177 (8.07)	1338 (7.20)
**Gender, n (%)**								
Male	549 (55.85)	6066 (51.02)	1302 (58.60)	12,337 (53.70)	1170 (61.39)	9297 (57.42)	1288 (58.73)	10,150 (54.58)
**BMI, n (%)**								
Unknown	39 (4.16)	1209 (10.13)	92 (4.14)	1848 (8.04)	57 (2.99)	830 (5.13)	65 (2.96)	947 (5.09)
< 18.5	81 (8.63)	563 (4.72)	56 (2.52)	537 (2.34)	44 (2.31)	479 (2.96)	69 (3.15)	680 (3.66)
18.5–24.9	325 (34.65)	4076 (34.15)	460 (20.70)	5133 (22.34)	379 (19.88)	3630 (22.42)	535 (24.40)	5542 (29.80)
25.0–29.9	201 (21.43)	2594 (21.73)	567 (25.52)	5879 (25.59)	493 (25.87)	4454 (27.51)	701 (31.97)	5950 (32.00)
> 30.0	292 (31.13)	3493 (29.27)	1047 (47.12)	9578 (41.69)	933 (48.95)	6799 (41.99)	823 (37.53)	5477 (29.45)
**DRG, Liver transplant (LT), Cystic fibrosis (CF), Heart failure (HF), n (%)**								
LT	9 (0.96)	9 (0.08)	68 (3.06)	49 (0.21)	65 (3.41)	69 (0.43)	15 (0.68)	20 (0.11)
CF	121 (12.90)	564 (4.73)	26 (1.17)	125 (0.54)	6 (0.31)	39(.24)	0 (0.00)	3 (0.02)
HF	6 (0.64)	19 (0.16)	45 (2.03)	127 (0.55)	36 (1.89)	121 (0.75)	62 (2.83)	277 (1.49)
**CCS, Nutritional deficiencies (ND), Esophageal disorders (ED), Essential hypertension (EH), n (%)**								
ND	193 (20.58)	1040 (8.71)	203 (9.14)	1844 (8.03)	156 (8.18)	1416 (8.75)	172 (7.84)	1721 (9.25)
ED	112 (11.94)	585 (4.90)	228 (10.26)	2283 (9.94)	239 (12.54)	1959 (12.10)	271 (12.36)	2551 (13.72)
EH	104 (11.09)	886 (7.42)	648 (29.16)	5834 (25.39)	825 (43.28)	5968 (36.86)	1053 (48.02)	8715 (6.86)
**MED, Tazobactam (T), Vancomycin (V), Acetaminophen (A), n (%)**								
T	403 (42.96)	2089 (17.50)	791 (35.60)	3742 (16.29)	567 (29.75)	2662 (16.44)	515 (23.48)	2892 (15.55)
V	366 (39.02)	1936 (16.22)	762 (34.29)	4485 (19.52)	620 (32.53)	3656 (22.58)	655 (29.87)	489 (22.53)
A	755 (80.49)	9883 (82.81)	1816 (81.73)	19,782 (86.10)	1585 (83.16)	14,097 (87.06)	1889 (86.14)	16,601 (89.27)
**Onset time, days, median [interquartile range]**								
Days	3 [2–6]	–	3 [2–5]	–	3 [2–6]	–	3 [2–6]	–

Note: *AKI* Acute kidney injury, *Non-AKI* Not acute kidney injury. Values for categorical variables are given as number (percentage), AKI onset time is given as median [interquartile range] of admission days

**Fig. 1:**
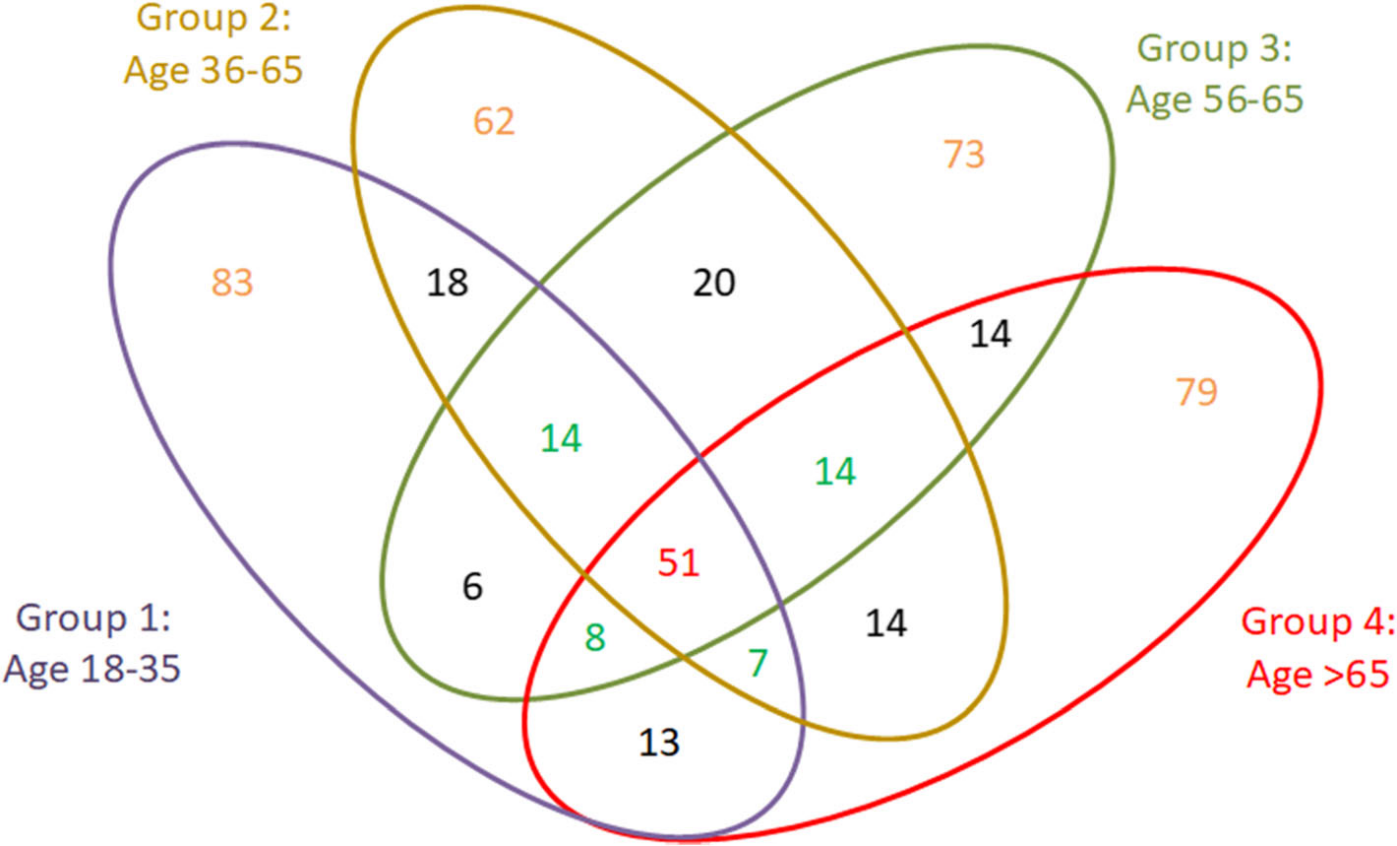
Venn diagram for the top 200 features identified in four age groups. This figure shows the number of overlapping features identified as top 200 across the four age groups

**Fig. 2:**
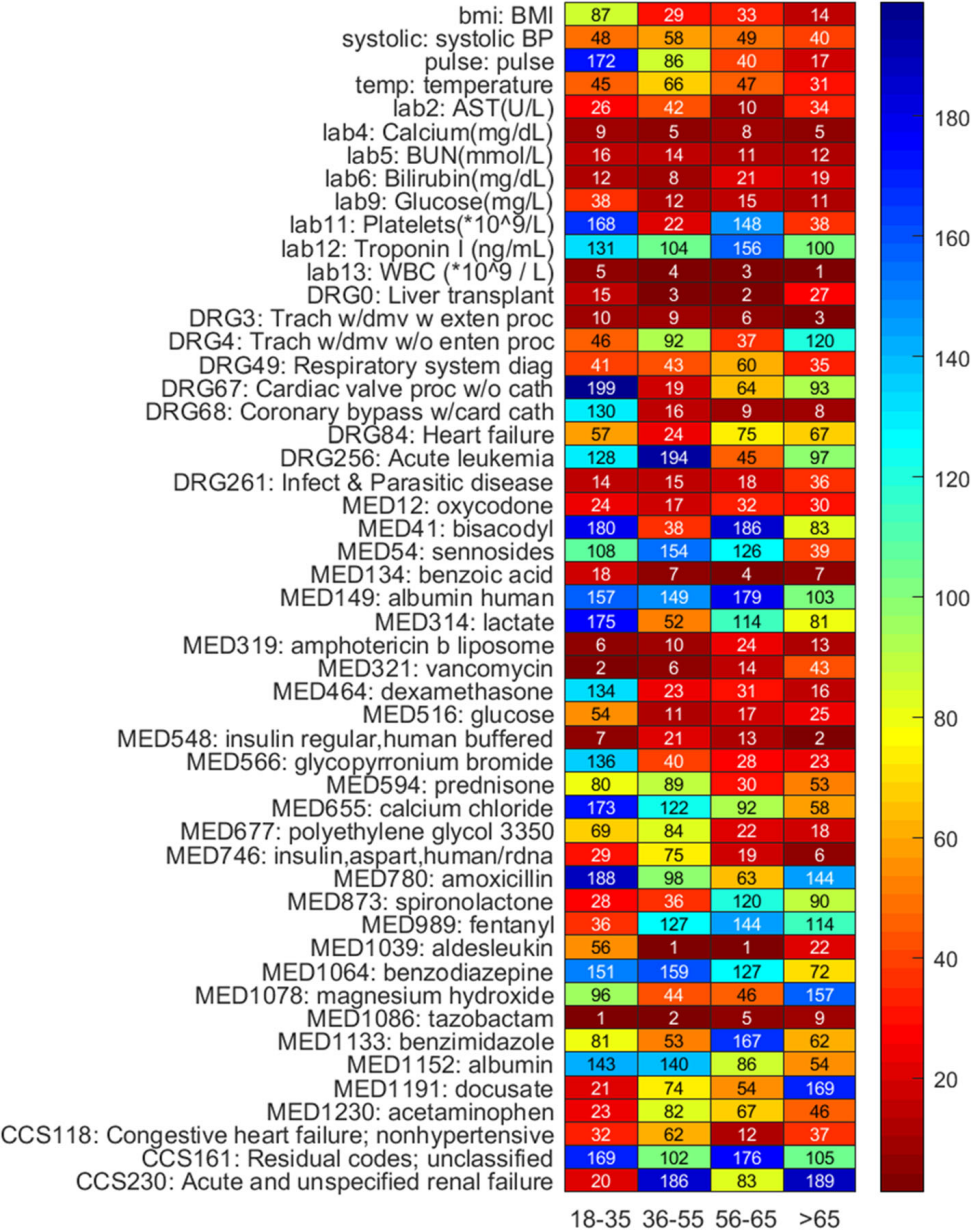
Heat map of the top-200 risk factors appeared in all four age groups. The figure shows the corresponding ranking of each factor in the GBM model

Figure 1 is the Venn diagram of the top 200 risk factors identified for each of the four age groups obtained by the GBM algorithm and shows the number of overlapping factors identified across strata. Figure 2 shows the common factors that appear in the top 200 important risk factor list across all four age groups. The variable importance plots for the top-ranked features for predicting AKI across age strata are shown in Supplementary Fig. S1, which illustrated an exponential decline trend in the contribution of top-*k* variables to AUROC gain. Moreover, overlapping factors in the top 200 list across only three age groups are shown in Supplementary Fig. S2.

**Fig. 3:**
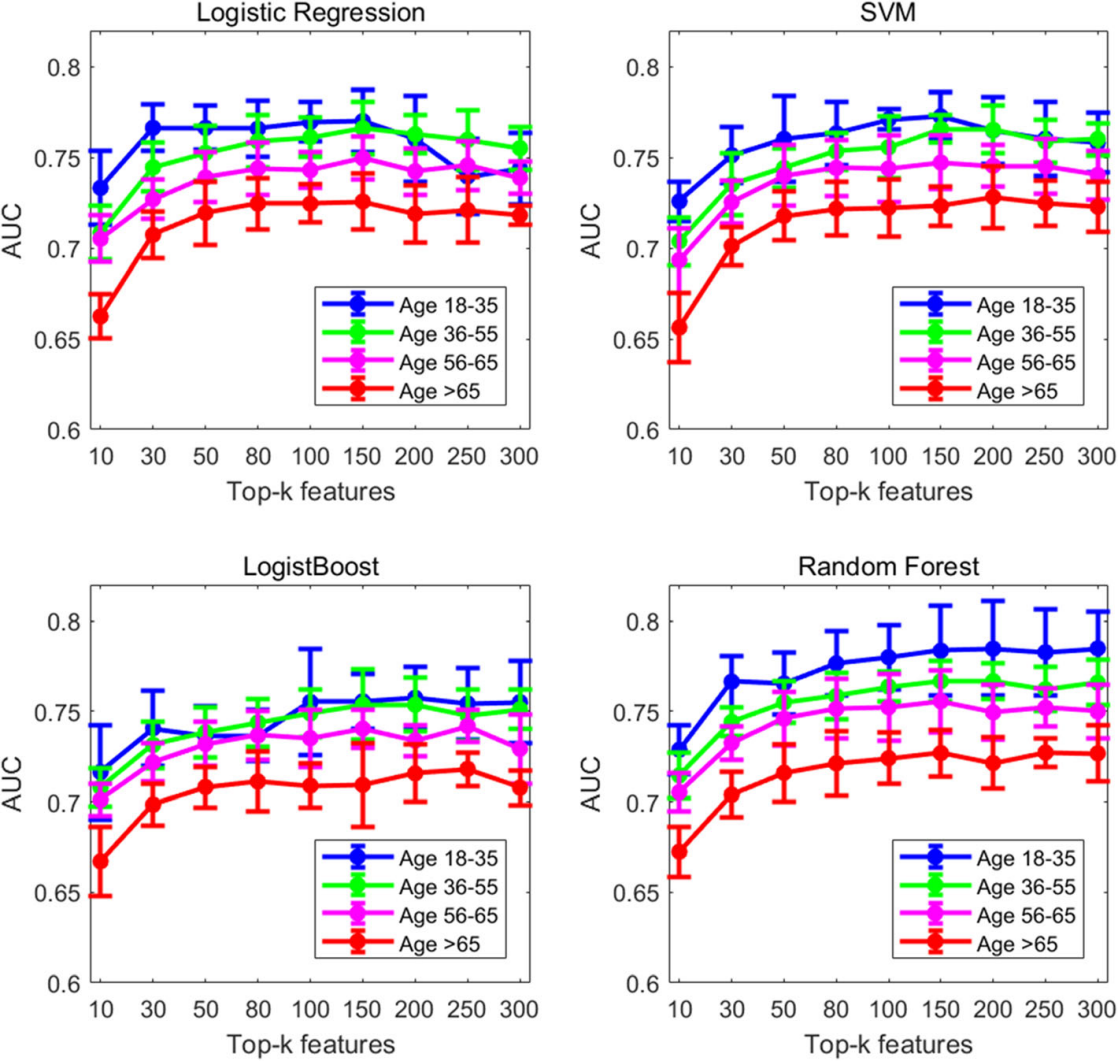
Prediction trends of the top-ranking features with under-sampling for the four age groups across different machine learning models

**Fig. 4:**
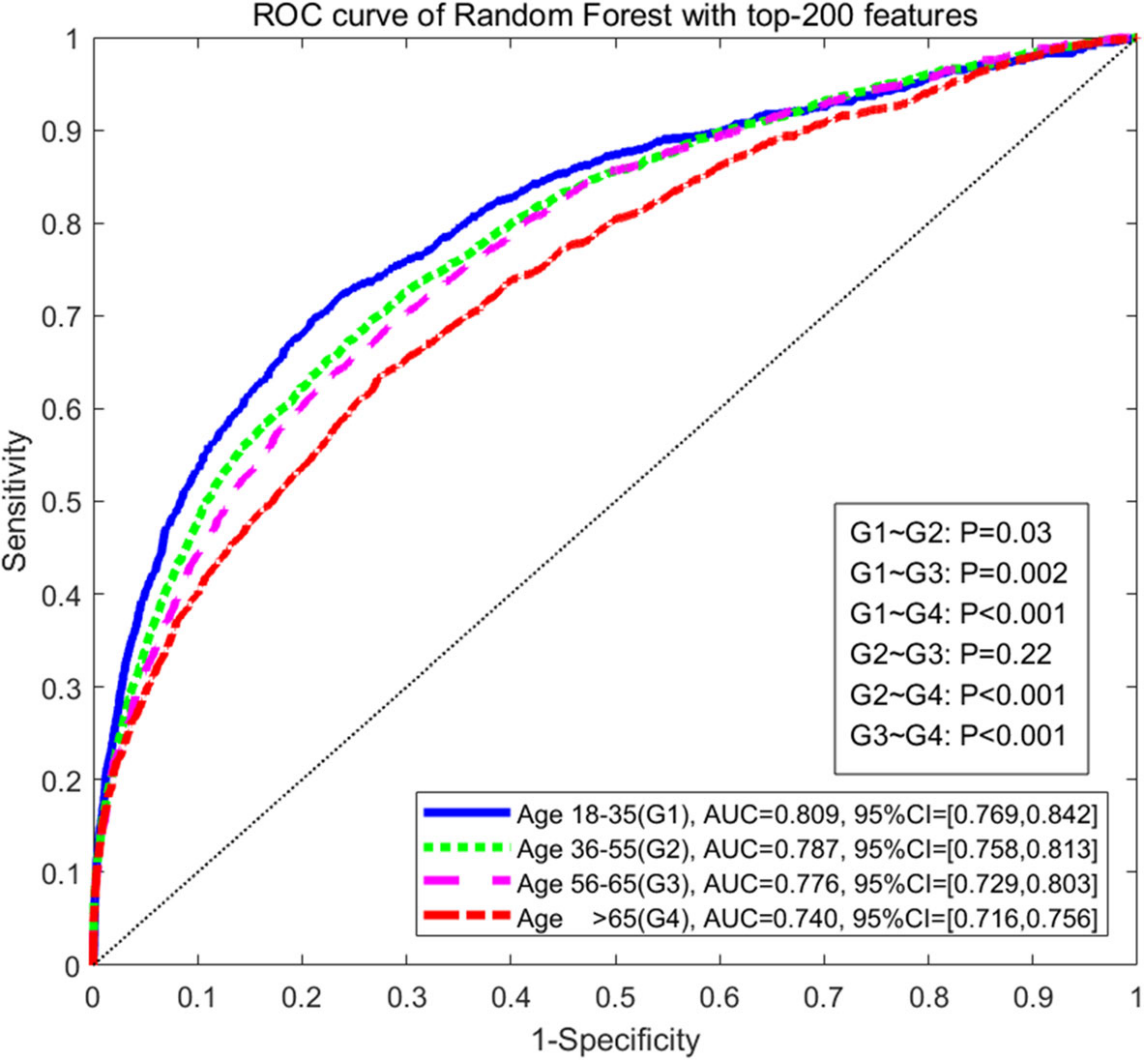
ROC curves of random forest without under-sampling for the four age groups

To further assess the discrimination of the top-ranked features for AKI prediction, we conducted a series of prediction experiments by including different numbers of top-*k* features (i.e. *k* = 10 to 300) in four machine learning models (i.e., logistic regression, SVM, LogitBoost, and random forest). Figure 3 shows the predictability trend of top-*k* important features with under-sampling of the majority class (please refer to Supplementary Fig. S3 for results from without under-sampling). Supplementary Table S4 illustrates the AUROC and corresponding 95% confidence interval values (CI) for AKI prediction with under-sampling using top-200 variables of four age groups based on four machine learning models. Supplementary Table S5 provides results for several predicted probability cutoffs for the final model and corresponding sensitivity, specificity, positive predictive value (PPV), and negative predictive value (NPV) in predicting AKI across age groups. Figure 4 shows the AUROC achieved by random forest using top-200 features without under-sampling for age groups 18–35, 36–55, 55–65, and > 65 years at 0.809 (95% CI, 0.769–0.842), 0.787 (95% CI, 0.758–0.813), 0.776 (95% CI, 0.729–0.803), and 0.740 (95% CI, 0.716–0.756) respectively. Above results demonstrated the predictability of AKI in the general inpatient population decreased as age increased, which may be due to more complex physiology of older adults. Table 3 shows that the significance levels of pairwise comparison of AKI incidence and prediction performance based on four machine learning methods between age groups, in which older patients showed a significantly higher incidence of AKI than younger age groups (*p* < 0.001), however the predictive power of the older group (i.e. > 65 age group) was significantly lower than that of other young groups (*p* < 0.05).

**Table 3:** Significant level of pairwise comparison

Age Group	G1 ~ G2	G1 ~ G3	G1 ~ G4	G2 ~ G3	G2 ~ G4	G3 ~ G4
Prevalence (%)	7.29 ~ 8.82	7.29 ~ 10.53	7.29 ~ 10.55	8.82 ~ 10.53	8.82 ~ 10.55	10.53 ~ 10.55
*P* value (prevalence)	**< 0.001**	**< 0.001**	**< 0.001**	**< 0.001**	**< 0.001**	0.97
AUC (LR)	0.759 [0.728–0.789] ~ 0.760 [0.750–0.770]	0.759 [0.728–0.789] ~ 0.746 [0.731–0.760]	0.759 [0.728–0.789] ~ 0.725 [0.713–0.736]	0.760 [0.750–0.770] ~ 0.746 [0.731–0.760]	0.760 [0.750–0.770] ~ 0.725 [0.713–0.736]	0.746 [0.731–0.760] ~ 0.725 [0.713–0.736]
*P* value (LR)	0.81	0.13	**< 0.001**	**0.022**	**< 0.001**	**0.004**
AUC (SVM)	0.767 [0.743–0.790] ~ 0.766 [0.753–0.780]	0.767 [0.743–0.790] ~ 0.742 [0.729–0.756]	0.767 [0.743–0.790] ~ 0.723 [0.702–0.744]	0.766 [0.753–0.780] ~ 0.742 [0.729–0.756]	0.766 [0.753–0.780] ~ 0.723 [0.702–0.744]	0.742 [0.729–0.756] ~ 0.723 [0.702–0.744]
*P* value (SVM)	0.10	**0.005**	**< 0.001**	0.12	**< 0.001**	0.06
AUC (LB)	0.750 [0.728–0.773] ~ 0.753 [0.733–0.772]	0.750 [0.728–0.773] ~ 0.738 [0.727–0.750]	0.750 [0.728–0.773] ~ 0.712 [0.701–0.724]	0.753 [0.733–0.772] ~ 0.738 [0.727–0.750]	0.753 [0.733–0.772] ~ 0.712 [0.701–0.724]	0.738 [0.727–0.750] ~ 0.712 [0.701–0.724]
*P* value (LB)	0.73	0.33	**0.002**	0.09	**< 0.001**	**0.009**
AUC (RF)	0.784 [0.769–0.800] ~ 0.766 [0.754–0.777]	0.784 [0.769–0.800] ~ 0.754 [0.741–0.768]	0.784 [0.769–0.800] ~ 0.723 [0.709–0.737]	0.766 [0.754–0.777] ~ 0.754 [0.741–0.768]	0.766 [0.754–0.777] ~ 0.723 [0.709–0.737]	0.754 [0.741–0.768] ~ 0.723 [0.709–0.737]
*P* value (RF)	0.11	**0.006**	**< 0.001**	0.11	**< 0.001**	**0.004**

*Abbreviation***:***AUC* the area under the receiver operating characteristic curve for top-200 features with under-sampling, *LR* Logistic Regression, *SVM* Support Vector Machine, *LB* LogistBoost, *RF* Random Forest, *G1* 18–35 age group; *G2* 36–55 age group, *G3* 56–65 age group, *G4* > 65 age group. *P* value in bold represents *p* < 0.05

## Discussion

Advanced age is an established independent risk factor for AKI [17], which may be due to the deterioration of renal function and the decrease in detoxification ability of drugs in the elderly [11, 26], making elderly highly sensitive to nephrotoxic drugs and susceptible to AKI. Findings from previous studies [11, 13, 27–30] support the proposition that age represents an important risk factor among the spectrum of risk factors for AKI. Although the incidence of AKI increases with age, we observed the predictability of AKI in the general inpatient population to decrease with age (Fig. 4). When comparing two data-sample processing mechanisms for addressing the imbalanced AKI vs non-AKI classification problem, namely with or without under-sampling, we consistently observed the predictive power of the four age-stratified models to decrease as age increased. The predictability of AKI risk in the older age group was significantly lower than that of the younger groups (*p* < 0.05).

Our research reached the same conclusions as Kane-Gill et al. [11]; however, our study had more patients (179,370 vs. 45,655) from all inpatient units (only ICU patients in Kane-Gill et al.), collected more clinical variables (1888 vs. 25), and achieved higher overall AUROCs. Moreover, the recent AKI prediction work by Google published in Nature [18] utilized EHR data from the U.S. Department of Veterans Affairs with over 700,000 patients and 366,856 distinct clinical variables, and their subgroup analysis on age showed lower AUROCs for patients in the older age groups which may also be due to patient heterogeneity.

To examine the change in relative risk of AKI predictors in the general inpatient population, we applied a machine learning-based feature selection algorithm over a large EMR dataset with close to two thousand variables to derive the relative ranking profiles and compared profiles across different age groups. Based on our previous research [31], we acknowledge that relative importance rankings of variables are affected by data samplings and feature selection methods. This study is not in any way to provide an absolute ranking of important predictors. It is to analyze and compare the relative variability and volatility of predictors between age groups using a single feature selection method (i.e., GBM). Figures 1 and 2 and Supplementary Fig. S2 illustrated the phenomenon that the relative risk of AKI predictors fluctuated between age groups.

Specifically, as shown in Table 2, low body mass index (BMI) was found to be associated with higher AKI risk in younger patients, but high BMI was found to be associated with higher AKI risk in elderly patients; and younger patients with cystic fibrosis, nutritional deficiencies or esophageal disorders have a higher risk of developing AKI compared with the older patients. Since efforts to quantify risk of AKI in older patients may be more difficult and older adults frequently have impaired drug clearance in addition to polypharmacy [26], clinical decision support systems to ensure proper drug usage and dosing in elderly may have special value. These findings implicate that AKI risk factors are heterogeneous, and age can modify the intensity of relationships between other factors and AKI. Therefore, future studies that evaluate risk factors needs to consider complex interactions between factors and their combinatorial effect on the outcome.

It is worth noting that feature selection method can identify factors with strong predictive ability, but these factors are not necessarily causal inducers. More specifically, some medicines by themselves do not increase risk for AKI, but the disease that is treated by the medicine increases the risk of AKI. The data we extracted is time-stamped with daily interval since admission, so the data granularity is coarse, and it is difficult to affirm whether a disease caused AKI or taking a medicine for an illness led to AKI. For example, in Supplementary Fig. S3, insulin (MED548) was identified as an important predictor of AKI in all age groups, and presumably this is just a marker for diabetes, i.e. patients with diabetes or diabetic nephropathy are at higher risk for AKI. Another example, polyethylene glycol 3350 (MED677, see Supplementary Fig. S3) is an osmotically acting laxative and its relative risk ranking for predicting AKI increases with age (namely, 69, 84, 22, 18 across the four age groups). However, we cannot clearly affirm in this case whether AKI was caused by the clinical indication that requires laxatives or because using a large amount of such laxatives would cause disturbance of water and electrolytes in the intestine, thereby inducing AKI. Thus, whether a drug increases patient risk for developing AKI requires rigorous demonstration from clinical experiments.

Furthermore, the granularity of medication data extraction and processing may not change prediction performance but will affect the knowledge learned by the machine learning models [32]. Considering the drug metabolism cycle, in this study, we only considered medications taken within a week, which would treat long-term (> 7 days) and short-term medication intakes the same. In recent years clinical studies have recognized that long-term use of drugs that inhibit gastric acid secretion (e.g., proton pump inhibitor [33, 34]) is likely to cause acute renal failure. Our model identified glycopyrronium bromide (MED566, see Supplementary Fig. S3) typically used for functional gastrointestinal disorders with an effect of inhibiting gastric secretion and regulating gastrointestinal motility, to have a higher relative risk ranking with respect to AKI that increased with age (namely, 136, 40, 28, 23 for four age groups). Hence, future work needs to consider length and amount of drug usage.

Several limitations in the present research must be considered. First, we limited the analysis to patients with a minimum eGFR (estimated glomerular filtration rate) of 60 ml/min/1.73m^2^ and normal serum creatinine on the day of admission at hospital admission. We acknowledge that patients with reduced eGFR have an increased risk of developing AKI; however, we made the decision to focus on hospital-acquired AKI. Second, to enhance machine learning model interpretability, our discretization of lab tests and vitals would lead to the loss of some information in data. Third, we did not include service unit as a risk factor and only selected certain lab tests based on previous literature for AKI prediction. Fourth, since our study was not limited to the ICU, we did not include urine output criteria as a predictor nor using it to define AKI. Fifth, our age stratification was not fine grained, for example patients > 65 years old were lumped into one category. Finally, although we utilized a large cohort observed for up to a decade, they only reflect the population of one academic medical center. Replicating this study in other institutions would generalize conclusions.

## Conclusion

In conclusion, we took advantage of a large EMR dataset and applied machine learning methods to analyze the changing relative risk and prediction performance of AKI across age strata. Analysis results demonstrate that (a) AKI risk increases with age, but the ability to predict AKI declines with age due to the increasing complexity of the patients; (b) the relative importance of clinical predictors in predicting hospital-acquired AKI fluctuates between age groups. The study findings suggest that accurate AKI risk prediction in elderly may require additional effort. It highlights the importance of considering age-specific risk differences in hospitalized patients to enhance AKI prevention in clinical care.

## Supplementary information

**Additional file 1: Table S1.** The KDIGO serum creatinine based staging system for acute kidney injury.

**Additional file 2: Table S2.** Discretization for patient vital signs.

**Additional file 3: Table S3.** The percentage of missing values in vital signs.

**Additional file 4: Table S4.** Prediction performance in terms of area-under-the-operating-characteristic-curve (AUROC) for model built with top-200 important features and under-sampling of majority class samples.

**Additional file 5: Table S5.** Sensitivity and specificity at different operating probability cutoffs for the random forest model prediction of acute kidney injury.

**Additional file 6: Figure S1.** Variable importance plot for top-ranked features across four age groups.

**Additional file 7: Figure S2.** Heat map of the top-200 important risk factors that appeared in only three age groups with the corresponding ranking of each factor in the GBM model.

**Additional file 8: Figure S3.** Prediction performance trend of different machine learning models learned with top-ranking features and without under-sampling of majority class samples across the four age groups.

**Additional file 9: Supplementary Method:** Gradient Boosting Machine (GBM).

## Data Availability

The clinical dataset used for analysis described in this study was obtained from the University of Kansas Medical Center (KUMC) HERON clinical data repository, which are not publicly available. Open reasonable request, amendment can be requested to the corresponding author to share the necessary data.

## Abbreviations

AKI: Acute kidney injury
EMR: Electronic medical records
KDIGO: Kidney Disease Improving Global Outcomes
AUROC: Area under the receiver operating characteristic curve
CKD: Chronic kidney disease
ICU: Intensive care units
eGFR: Estimated Glomerular Filtration Rate
SCr: Serum creatinine
MDRD: Modification of Diet in Renal Disease
HERON: Health Enterprise Repository for Ontological Narration
HIPAA: Health Insurance Portability and Accountability Act
Demo: Demographics
Lab: Lab tests
DRG: Admission diagnoses
CCS: Medical History
MED: Medications
GBM: Gradient boosting machine
SVM: Support vector machine
CI: Confidence interval values
PPV: Positive predictive value
NPV: Negative predictive value
