# Supplementary material for: Changing relative risk of clinical factors for hospital-acquired acute kidney injury across age groups: a retrospective cohort study
Source: BMC Nephrol. 2020 Aug 2;21:321. doi: 10.1186/s12882-020-01980-w (PMC7397647; doi:10.1186/s12882-020-01980-w)
Supplement: Supplementary file 1 — Additional file 1: Table S1. The KDIGO serum creatinine based staging system for acute kidney injury. [file 12882_2020_1980_MOESM1_ESM.docx]

**Table S1.** The KDIGO serum creatinine based staging system for acute kidney injury

| **AKI Stage** | **Serum Creatinine (SCr) Criteria** |
| --- | --- |
| 1 | Increase >26.4 µmol/L (0.3 mg/dL) with 48 hours or 1.5-1.9 times baseline within 7 days |
| 2 | Increase 2.0-2.9 times baseline |
| 3 | Increase creatinine >354 µmol/L (4.0 mg/dL) or 3 times baseline |
