# Supplementary material for: Changing relative risk of clinical factors for hospital-acquired acute kidney injury across age groups: a retrospective cohort study
Source: BMC Nephrol. 2020 Aug 2;21:321. doi: 10.1186/s12882-020-01980-w (PMC7397647; doi:10.1186/s12882-020-01980-w)
Supplement: Supplementary file 2 — Additional file 2: Table S2. Discretization for patient vital signs. [file 12882_2020_1980_MOESM2_ESM.docx]

**Table S2.** Discretization for patient vital signs

| Vitals | Categories |
| --- | --- |
| **BMI** | <18.5, [18.5-24.9], [25.0–29.9], >30.0, Unknown |
| **Diastolic BP** | <80, [80–89], [90–99], >100, Unknown |
| **Systolic BP** | <120, [120–139], [140–159], >160, Unknown |
| **Pulse** | <50, [50–65], [66–80], [81–100], >100, Unknown |
| **Temperature** | <95.0, [95.0–97.6], [97.7–99.5], [99.5–104.0], >104.0, Unknown |
