# Supplementary material for: Changing relative risk of clinical factors for hospital-acquired acute kidney injury across age groups: a retrospective cohort study
Source: BMC Nephrol. 2020 Aug 2;21:321. doi: 10.1186/s12882-020-01980-w (PMC7397647; doi:10.1186/s12882-020-01980-w)
Supplement: Supplementary file 3 — Additional file 3: Table S3. The percentage of missing values in vital signs. [file 12882_2020_1980_MOESM3_ESM.docx]

**Table S3.** The percentage of missing values in vital signs

| Vitals  N (%) | Age 18-35 | Age 36-55 | Age 56-65 | Age >65 |
| --- | --- | --- | --- | --- |
| **BMI** | 3588(27.87) | 2350(9.33) | 3548(19.60) | 1896(9.12) |
| **Diastolic BP** | 54(0.42) | 47(0.19) | 0(0) | 0(0) |
| **Systolic BP** | 54(0.42) | 47(0.19) | 0(0) | 0(0) |
| **Pulse** | 54(0.42) | 46(0.18) | 1(0) | 1(0) |
| **Temperature** | 94(0.73) | 67(0.27) | 29(0.16) | 19(0.09) |
