# Supplementary material for: Changing relative risk of clinical factors for hospital-acquired acute kidney injury across age groups: a retrospective cohort study
Source: BMC Nephrol. 2020 Aug 2;21:321. doi: 10.1186/s12882-020-01980-w (PMC7397647; doi:10.1186/s12882-020-01980-w)
Supplement: Supplementary file 4 — Additional file 4: Table S4. Prediction performance in terms of area-under-the-operating-characteristic-curve (AUROC) for model built with top-200 important features and under-sampling of majority class samples. [file 12882_2020_1980_MOESM4_ESM.docx]

**Table S4.** Prediction performance in terms of area-under-the-operating-characteristic-curve (AUROC) for model built with top-200 important features and under-sampling of majority class samples

| **Age group** | **Logistic regression** | **SVM** | **LogitBoost** | **Random forest** |
| --- | --- | --- | --- | --- |
| **18-35** | 0.759*  (0.728-0.789) | 0.767*  (0.743-0.790) | 0.750*  (0.728-0.773) | **0.784**  **(0.769-0.800)** |
| **36-55** | 0.760  (0.750-0.770) | 0.765  (0.753-0.779) | 0.753*  (0.733-0.772) | **0.766**  **(0.754-0.777)** |
| **56-65** | 0.746  (0.731-0.760) | 0.742  (0.729-0.756) | 0.738*  (0.727-0.750) | **0.754**  **(0.741-0.768)** |
| **>65** | 0.723  (0.713-0.736) | 0.723  (0.702-0.744) | 0.712  (0.701-0.724) | **0.723**  **(0.709-0.737)** |
| **Note:** * represents the method in bold significantly improves performance compared with this corresponding method (p<0.05). | | | | |
