# Supplementary material for: Changing relative risk of clinical factors for hospital-acquired acute kidney injury across age groups: a retrospective cohort study
Source: BMC Nephrol. 2020 Aug 2;21:321. doi: 10.1186/s12882-020-01980-w (PMC7397647; doi:10.1186/s12882-020-01980-w)
Supplement: Supplementary file 5 — Additional file 5: Table S5. Sensitivity and specificity at different operating probability cutoffs for the random forest model prediction of acute kidney injury. [file 12882_2020_1980_MOESM5_ESM.docx]

**Table S5.** Sensitivity and specificity at different operating probability cutoffs for the random forest model prediction of acute kidney injury

| **Probability Cutoff** | **No. of Patients**­^a^ | **Sensitivity**  **(%)** | **Specificity**  **(%)** | **Positive Predictive Value (%)** | **Negative Predictive Value (%)** |
| --- | --- | --- | --- | --- | --- |
| **Age Group 1** | | | | | |
| $\geq$0.03 | 8500 | 91.47 | 35.97 | 10.09 | 98.17 |
| $\geq$0.05 | 5926 | 84.54 | 56.99 | 13.38 | 97.91 |
| $\geq$0.08 | 3794 | 73.45 | 73.98 | 18.16 | 97.26 |
| $\geq$0.10 | 2905 | 67.27 | 80.95 | 21.72 | 96.92 |
| $\geq$0.20 | 924 | 39.23 | 95.34 | 39.83 | 95.23 |
| **Age Group 2** | | | | | |
| $\geq$0.03 | 20521 | 96.04 | 19.97 | 10.40 | 98.12 |
| $\geq$0.05 | 15586 | 89.47 | 40.81 | 12.76 | 97.57 |
| $\geq$0.08 | 10163 | 77.32 | 63.24 | 16.90 | 96.65 |
| $\geq$0.10 | 7713 | 69.58 | 73.16 | 20.04 | 96.13 |
| $\geq$0.20 | 2062 | 35.82 | 94.49 | 38.60 | 93.84 |
| **Age Group 3** | | | | | |
| $\geq$0.03 | 15775 | 97.53 | 14.06 | 11.78 | 97.98 |
| $\geq$0.05 | 13259 | 93.13 | 29.08 | 13.39 | 97.29 |
| $\geq$0.08 | 9632 | 85.52 | 50.58 | 16.92 | 96.74 |
| $\geq$0.10 | 7576 | 76.92 | 62.27 | 19.35 | 95.82 |
| $\geq$0.20 | 1997 | 38.77 | 92.23 | 37.01 | 92.75 |
| **Age Group 4** | | | | | |
| $\geq$0.03 | 19246 | 98.63 | 8.14 | 11.24 | 98.06 |
| $\geq$0.05 | 16535 | 92.93 | 22.04 | 12.33 | 96.36 |
| $\geq$0.08 | 11791 | 82.17. | 46.28 | 15.28 | 95.65 |
| $\geq$0.10 | 8940 | 73.19 | 60.56 | 17.95 | 95.04 |
| $\geq$0.20 | 1744 | 31.10 | 94.29 | 39.11 | 92.07 |
| ^a^ Represents the total number of patients who achieved a probability cutoff greater than or equal to value in column 1 during their admission. | | | | | |
