# Supplementary figures and images for: Changing relative risk of clinical factors for hospital-acquired acute kidney injury across age groups: a retrospective cohort study
Source: BMC Nephrol. 2020 Aug 2;21:321. doi: 10.1186/s12882-020-01980-w (PMC7397647; doi:10.1186/s12882-020-01980-w)

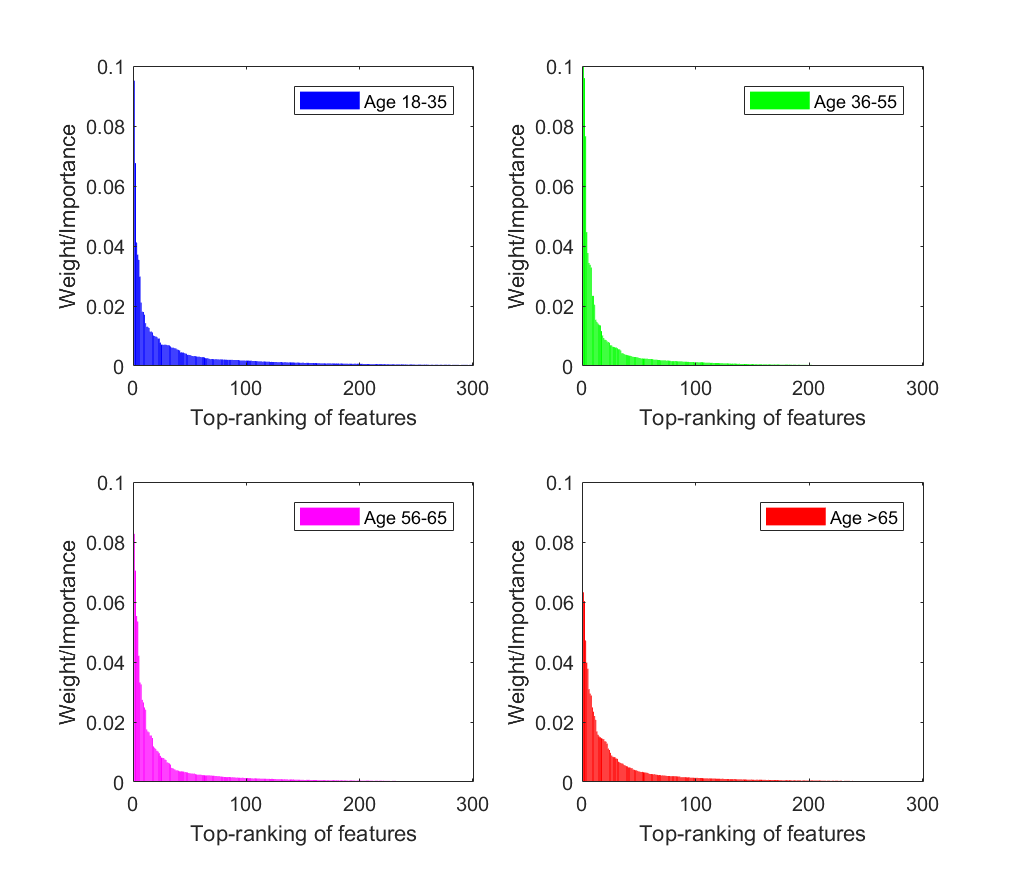

Supplement: Supplementary file 6 — Additional file 6: Figure S1. Variable importance plot for top-ranked features across four age groups. [file 12882_2020_1980_MOESM6_ESM.png]

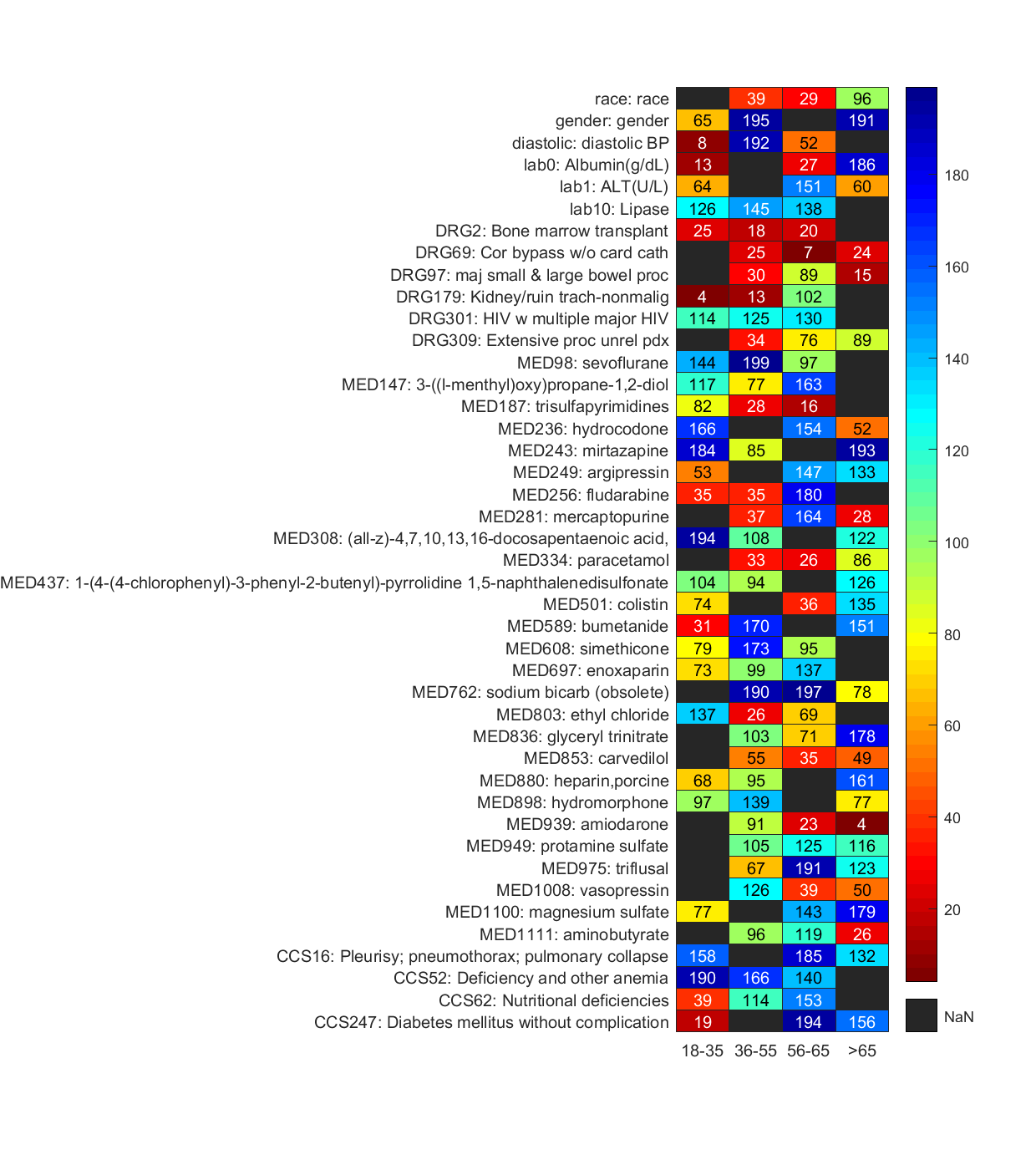

Supplement: Supplementary file 7 — Additional file 7: Figure S2. Heat map of the top-200 important risk factors that appeared in only three age groups with the corresponding ranking of each factor in the GBM model. [file 12882_2020_1980_MOESM7_ESM.png]

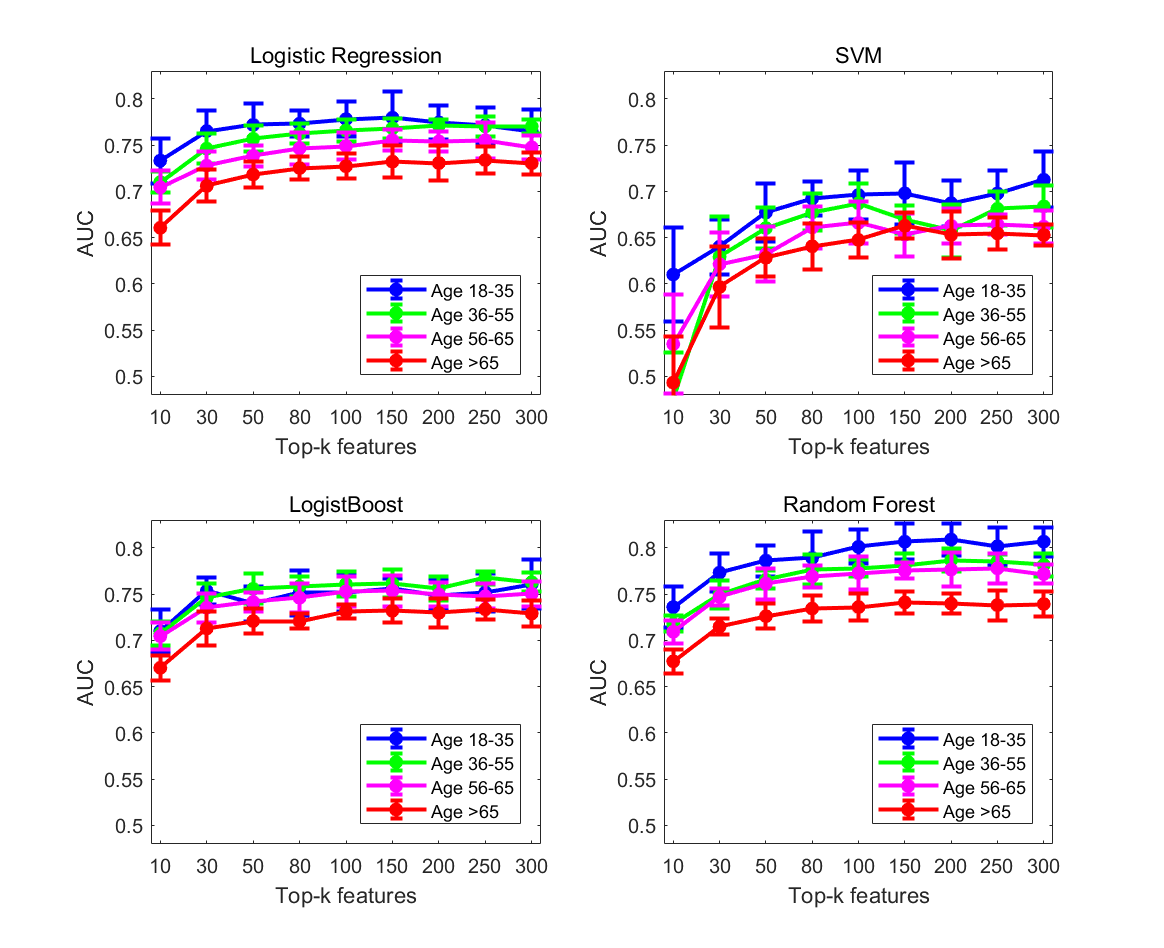

Supplement: Supplementary file 8 — Additional file 8: Figure S3. Prediction performance trend of different machine learning models learned with top-ranking features and without under-sampling of majority class samples across the four age groups. [file 12882_2020_1980_MOESM8_ESM.png]
