# Supplementary material for: Changing relative risk of clinical factors for hospital-acquired acute kidney injury across age groups: a retrospective cohort study
Source: BMC Nephrol. 2020 Aug 2;21:321. doi: 10.1186/s12882-020-01980-w (PMC7397647; doi:10.1186/s12882-020-01980-w)
Supplement: Supplementary file 9 — Additional file 9: Supplementary Method: Gradient Boosting Machine (GBM). [file 12882_2020_1980_MOESM9_ESM.docx]

**Supplementary Method:** Gradient Boosting Machine (GBM)

Gradient boosting machine model allows the development of a predictive model by first creating a single decision tree that best identifies patients with AKI risk, then creates a subsequent decision tree that identifies patients developing AKI that cannot be accurately predicted through an iterative process, each of which is designed to better predict AKI cases that were missed in early trees. Therefore, the final model consists of a series of trees, with the optimal number of trees, depth and learning rate determined by a ten-fold cross-validation in the derived data set. In this algorithm, variable importance is determined by calculating the relative influence of each variable: whether that variable was selected to split on during the tree building process, and how much the squared error (over all trees) improved (decreased) as a result. Meantime, its superior performance has been demonstrated by Koyner et al.^1^ on AKI prediction and our team’s research on AKI feature selection^2^.

1. Koyner JL, Carey KA, Edelson DP, Churpek MM: The Development of a Machine Learning Inpatient Acute Kidney Injury Prediction Model. *Crit. Care Med.* 46: 1070–1077, 2018

2. Wu L, Hu Y, Liu X, Zhang X, Chen W, Yu ASL, Kellum JA, Waitman LR, Liu M: Feature Ranking in Predictive Models for Hospital-Acquired Acute Kidney Injury. *Sci. Rep.* [Internet] 8: 17298, 2018 Available from: https://doi.org/10.1038/s41598-018-35487-0.
